# Designing and understanding light-harvesting devices with machine learning

**DOI:** 10.1038/s41467-020-17995-8

**Published:** 2020-09-11

**Authors:** Florian Häse, Loïc M. Roch, Pascal Friederich, Alán Aspuru-Guzik

**Affiliations:** 1 Department of Chemistry and Chemical Biology, Harvard University, 12 Oxford Street, Cambridge, 02138 MA USA; 2 CIFAR AI Chair, Vector Institute for Artificial Intelligence, 661 University Avenue, Toronto, ON M5S 1M1 Canada; 3 Department of Computer Science, University of Toronto, 214 College Street, Toronto, ON M5S 3H6 Canada; 4 Chemical Physics Theory Group, Department of Chemistry, University of Toronto, 80 St. George Street, Toronto, ON M5S 3H6 Canada; 5 ChemOS Sàrl, Lausanne, VD 1006 Switzerland; 6 Institute of Nanotechnology, Karlsruhe Insititute of Technology, Hermann-von-Helmholtz-Platz 1, 76344 Eggenstein-Leopoldshafen, Germany; 7 Lebovic Fellow, Canadian Institute for Advanced Research (CIFAR), 661 University Avenue, Toronto, ON M5S 1M1 Canada

**Keywords:** Light harvesting, Energy transfer, Excited states, Statistics

## Abstract

Understanding the fundamental processes of light-harvesting is crucial to the development of clean energy materials and devices. Biological organisms have evolved complex metabolic mechanisms to efficiently convert sunlight into chemical energy. Unraveling the secrets of this conversion has inspired the design of clean energy technologies, including solar cells and photocatalytic water splitting. Describing the emergence of macroscopic properties from microscopic processes poses the challenge to bridge length and time scales of several orders of magnitude. Machine learning experiences increased popularity as a tool to bridge the gap between multi-level theoretical models and Edisonian trial-and-error approaches. Machine learning offers opportunities to gain detailed scientific insights into the underlying principles governing light-harvesting phenomena and can accelerate the fabrication of light-harvesting devices.

## Abstract (web summary)

Photon-induced charge separation phenomena are at the heart of light-harvesting applications but challenging to be described by quantum mechanical models. Here the authors illustrate the potential of machine-learning approaches towards understanding the fundamental processes governing electronic excitations.

## Introduction

Converting sunlight into energy is an essential metabolic step for many organisms and thus one of the key fundamental processes driving life on Earth. The abundance of solar power, and the fact that plants can leverage photochemical processes to convert it into chemical energy, provides the opportunity to use it as a massive renewable energy resource^1^. Indeed, the energy provided by the sun is expected to be sufficient to satisfy the worldwide energy consumption^2^. As such, developing scalable, cost-efficient systems to harness solar energy offers a roadmap to approach some of the key societal challenges of the 21st century, including the development of sustainable clean energy technologies^3^. The key to viable artificial light-harvesting systems are operations at high power conversion efficiencies with long life times and low production costs.

Biological organisms capable of producing chemical energy from sunlight, a process catalyzed by photon-induced charge separation, inspire the design of artificial light-harvesting devices for various applications: photovoltaic systems create electrical voltage and current upon photon absorption^4^, excitonic networks are developed for efficient excitation energy transport^5^, and functional materials powered by sunlight enable carbon dioxide (CO_2_) reduction^6^ and water splitting^7^, to name a few. While artificial solar energy conversion is on the rise, current technologies need to be advanced to expedite the transition to a net-zero carbon economy. Detailed mechanistic understanding and structural insights into the physiological processes in biological organisms to harvest sunlight could inspire the design of artificial light-harvesting devices.

**Fig. 1:**
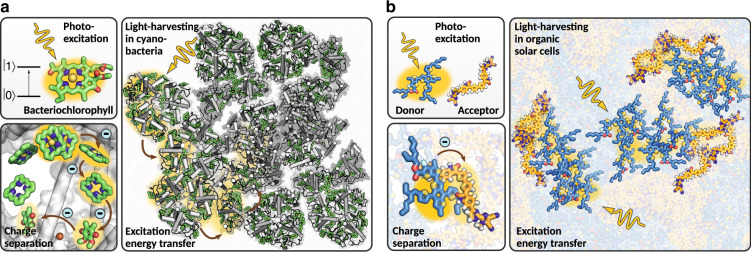
Light harvesting in phototrophic organisms and organic solar cells. **a** Variants of the chlorophyll pigment molecules can create excitons upon photon absorption, which are transferred to the reaction center for charge separation. **b** Excitons created upon photon absorption by the donor material are transferred to the donor–acceptor interface for charge separation.

Over millions of years, photoautotrophs, notably cyanobacteria and plants, have developed efficient and robust strategies to achieve direct solar-to-fuel conversion with photosynthesis. In this process, chemical energy is produced in the form of carbohydrate molecules, e.g. sugars, which are synthesized from water and CO_2_. These reactions are driven by the absorption of photons collected from sunlight. The primary steps of natural photosynthesis involve the creation of spatially separated electron-hole pairs upon photon absorption in the light-dependent reactions. The resulting electric potential drives the oxidation of water to oxygen in the light-independent reactions. This water-splitting process is at the heart of the energetics of photosynthesis^1^. The key processes of the light-dependent reactions are facilitated by self-assembled light-harvesting pigment–protein complexes at high energy conversion efficiencies and robustness^4^: the formation of electronic excitations induced by photon absorption as the primary energy conversion step, followed by excitation energy transport (EET) and finally charge-separation, via charge-transfer (CT) excitations, to drive chemical reactions (see Fig. 1a).

With optimal environmental conditions, photosynthetic organisms can convert almost all absorbed photons into stable photoproducts^8^ and thus operate at nearly 100% quantum efficiency^9^. However, solar energy conversion efficiency must ultimately be assessed from the perspective of complete life cycles^10^. While artificial light-harvesting devices can achieve almost as high quantum efficiencies^11^, their overall power conversion efficiency is typically higher than those of photosynthetic organisms, which typically does not exceed 1% for crop plants^12^, and 3% for microalgae^13^. Indeed, photosynthetic organisms are more concerned about survival (i.e. fitness) than high biomass production (i.e., growth). Adverse, rapid changes in the incident photon flux are accounted for by small structural changes of one or more of the light-harvesting proteins to open up energy dissipation pathways and thus limit the formation of harmful photoproducts such as reactive oxygen species^14^. For this reason, the vital property of the photosynthetic apparatus is functional robustness despite constantly fluctuating environments (i.e., disorder) on all levels of organization. Solar technologies based on low-cost molecular materials such as polymers, organic semiconductors, and nanoparticles face similar challenges of fluctuating environments and phototoxicity and may benefit greatly when steered by the design principles on which natural photosynthesis is operating.

Designing light-harvesting devices requires a well-founded understanding of the emergence of macroscopic materials properties from their microscopic structures. Computational models can help to unveil these structure-property relations and thus accelerate the targeted development of artificial light-harvesting systems. However, challenges of the current computational models lie in the computational cost associated with the quantum mechanical treatment of the relevant mechanisms dominated by EET and CT events as well as material degradation pathways. Established theoretical descriptions to quantify these processes are under active development, but are oftentimes computationally involved, and sometimes cover only a subset of the phenomena relevant in experimental settings. In fact, one of the outstanding challenges in computational materials science consists in closing the gap between the length-scales of single molecules and macroscopic materials as well as the time-scales, bridging ultrafast electronic events to slow collective nuclear motions^15^. In many cases, only empirical models are available to approximately describe structure-property relations.

Artificial intelligence (AI), notably machine learning (ML), has experienced rising interest by the scientific community^16–20^. Recent progress in the field of AI allows to rethink current approaches and design methods with accuracies comparable to state-of-the-art theoretical models at a fraction of the computational cost. ML, as a subdiscipline of AI, presents a particularly promising approach to this endeavor. By identifying patterns in data, ML can leverage statistical correlations—in contrast to the laws of physics—to predict the properties of the system of interest. Such transformative phenomenological models have the potential to accelerate scientific discovery^21–24^.

In this perspective, we outline recent successes of ML to drive the scientific understanding of phenomena and applications of light-harvesting. Specifically, we highlight the benefits of ML to complement quantum mechanical models for estimating EET and CT properties, as well as the opportunities to predict macroscopic device properties, notably related to device stability, directly from microscopic structures. Despite these successes, there are promising new venues to explore and to leverage from ML approaches, which are currently explored by the community, as overviewed hereafter. We conclude this perspective by spotlighting potential applications to foster a deeper integration of ML into established scientific workflows to tackle today’s energy challenge at a faster pace.

## Computational models for light-harvesting

From photon capture to charge transfer, quantum mechanical phenomena are at the heart of the fundamental processes governing photosynthesis. Full theoretical descriptions and detailed understanding of these processes are most desirable to derive roadmaps for the design of artificial light-harvesting devices. Over the last decade, EET and CT events in large photosynthetic complexes, such as the light-harvesting complex II (LHII) or the Fenna–Matthews–Olson (FMO) complex, have been a topic of interest from both a theoretical and experimental perspective^25–27^. However, full quantum mechanical treatments of all degrees of freedom in these molecular systems is computationally infeasible due to their large sizes (exceeding 100,000 atoms) and the relevant time-scales ranging from ultrafast electronic processes (fs to ps) to slow reorganization events (μs to ms)^28^.

Computational models to study EET and CT excitations in biological light-harvesting complexes rely on hybrid quantum mechanics/molecular mechanics (QM/MM) simulations, where the electronic structure of a subsystem, typically the molecular pigments, is modeled quantum mechanically while the surrounding bath, e.g. the protein scaffold or the solvent, is described by a classical force field. Molecular excitations are commonly assumed to be mostly governed by excitations between the ground and the first excited states, and are modulated by thermal fluctuations in the nuclear geometry of the pigments and their surroundings. Although non-adiabatic excited state dynamics calculations could reveal the EET processes, only reduced models which treat the dynamics of the bath implicitly, can currently be afforded to study the governing principles of photosynthesis.

In a first approximation, excitation energy correlation functions can be determined from low-level quantum chemistry methods for estimating excitation energies of molecular pigments in conformations generated with classical molecular dynamics^29,30^. Extensions to this approach include quantum mechanical corrections to molecular geometries at increased computational costs^31^, or ground state dynamics calculations based on density functional theory (DFT)^32^. In a second step, EET properties are determined via open quantum system dynamics schemes. Numerically accurate approaches that account for the non-Markovian transfer process, e.g. the hierarchical equations of motion (HEOM)^33,34^, are computationally demanding, and can only be afforded for selected systems.

Although bio-inspired design of molecules and materials for light-harvesting applications has been of interest for decades^35^, the computational cost for describing EET and CT excitation events with aforementioned theoretical models poses major challenges to large-scale ab initio studies. Computational descriptions of solar cells, for example, require elaborate and costly multi-scale models. Solar cells constitute devices which, inspired by the light-dependent reactions of photosynthesis, convert sunlight into electrical energy by generating spatially separated electron-hole pairs upon photon absorption. Several device architectures have been proposed for solar cells, differing in their material constitutions and compositions^36^. The efficiency of the light-to-energy conversion process is determined by the electronic properties of the constituting materials that regulate photon absorption and exciton dissociation events.

In the case of inorganic solar cells, charge separation is a spontaneous process. Most of the commercially available first-generation solar cells are based on pn-junctions created by doped polycrystalline or single-crystal silicon^36^. Second-generation thin film solar cells include cadmium telluride (CdTe)^37,38^ and copper indium gallium selenide (CIGS) technologies^39^. Recently, perovskite solar cells (PSCs)^40,41^ have experienced increased attention as breakthroughs in materials and device architectures boosted their efficiencies and stabilities^42^. PSCs are typically composed of inorganic lead halide matrices, and contain inorganic or organic cations. Power conversion in PSCs is achieved by the direct absorption and conversion of incoming photons into free electrons and holes which are then extracted through p- and n-type contacts.

Organic solar cells (OSCs) constitute another class of solar cell technologies that uses phase-separated mixtures of two or more materials in a bulk-heterojunction architecture to absorb light and split the exciton into electron-hole pairs at the interface between the two (or three) materials (see Fig. 1b)^43,44^. Thus, OSCs fall somewhere between the limits of photosynthesis and crystalline solar-cell materials with desirable properties often limited by energetic and structural disorder^4,45,46^. They have a number of appealing advantages over their inorganic counterparts, such as mechanical flexibility, lower energy payback time, being free of heavy metals and they can be successfully stabilized. Early OSCs have been proposed with fullerenes as acceptor materials due to their excellent electron-transporting properties and favorable bulk heterojunction morphology^47,48^. However, fullerene-based OSCs present critical limitations related to fundamentally constrained energy levels^49^, and photochemical instability^50^. In fact, the efficiency of organic solar cells based on fullerene derivatives as acceptor materials was limited to <12% and mostly saturated between 2012 and 2017^36^. A major advance in engineering efficient OSC candidates was the discovery of several families of non-fullerene acceptor molecules^51,52^. Currently, these acceptors replace C_60_/C_70_ derivatives in all highly-efficient organic solar cells^44,53^, reaching power conversion efficiencies well beyond the highest PCEs achieved with fullerene-based acceptors. The large number of degrees of freedom arising from the complex aromatic structures allows to fine tune their electronic properties such as the optical gap, exciton diffusion length, exciton binding energy, the energy level alignment between the donor and acceptor materials, or the charge-carrier mobility. Further development is required to make OSCs based on non-fullerene acceptors ready for commercial applications, mostly to make non-fullerene acceptors chemically less complex and thus cheaper to produce on a large scale.

Computational tools employed to determine these properties often balance accuracy and computational cost. Time-dependent density functional theory (TD-DFT) is nowadays a widely used quantum chemical method to study excited states, notably due to its relatively low computational demands. However, errors from TD-DFT calculations can be significant particularly for large organic chromophores, and are highly influenced by the parametrization of the exchange-correlation functional^54,55^. A complementary approach to the computation of excitation properties relies on the Green’s function formalism to derive first-principles GW-BSE (Bethe-Salpeter)^56^, based on a one-electron Green’s function, *G*, and a screened Coulomb potential, *W*. GW constitutes a quasi-particle many-body theory, which is known to accurately estimate electronic excitations described by electron addition and removal processes^57^, as has been shown, for example, for corannulene-based materials^58,59^, organic molecules for photovoltaics^60^ and fullerene-porphyrin complexes^61^.

One known phenomenon which is particularly challenging to describe theoretically or observe experimentally is the reduction of open-circuit voltage in organic solar cells due to non-radiative decays that adversely affect the efficiency of solar-cell devices^62^. In the absence of complete and tractable theoretical models, the identification of empirical evidence for this hypothesis requires a lot of effort. For instance, Vandewal and coworkers have presented evidence for a universal relation between non-radiative decay rates of molecules used in OSCs and losses in open-circuit voltage and thus in power conversion efficiency^62^. The challenge in finding the relation between molecular structure and non-radiative decay rates consists in the fact that non-radiative decay is closely coupled to molecular vibrations and electron–phonon interactions. These phenomena are non-trivial to describe with ground-state methods based on the Born-Oppenheimer approximation such as DFT. The complexity of device architectures and the large number of materials properties, which determine device efficiencies and stabilities pose major obstacles to the microscopic understanding of macroscopic properties. Designing and engineering promising device candidates, therefore, remains to be a challenge and faster and more accurate computational tools are needed for in silico studies of solar cell efficiencies and stabilities^63^.

## Advances in machine learning

ML is emerging as a promising tool to accelerate resource-demanding computations and experiments in the physical sciences^16–20^. ML, as a subdiscipline to AI, generally encompasses algorithmic systems and statistical models capable of performing defined tasks without being provided specific instructions. Instead, ML models infer task-relevant information from provided data and thus learn how to solve these tasks. To this end, ML seeks to provide knowledge to computers through data that encodes observations and interactions with the world or parts of it^64^.

The ability of ML models to identify and exploit statistical correlations from examples offers opportunities in the physical sciences. ML has the potential to bridge the gap between the construction of elaborate and costly multi-theory models and resource demanding Edisonian trial-and-error approaches^65^. This offers opportunities to leverage ML, for example, to speculate about the performances of hypothetical, not yet fabricated OPV devices or materials based on measurements on other devices collected in the past. ML could, therefore, inspire the formulation of hypotheses, design principles, and scientific concepts.

The versatile applicability of AI in the sciences has already been realized decades ago. One of the earliest applications has been introduced with the Dendral and Meta-Dendral programs, which sought to develop an artificial expert level agent to determine molecular structures of unknown compounds from mass spectrometry data^66^. The Dendral initiative had first been launched in 1965 with the ambition to automate the decision-making process of organic chemists^67^ and many computational tools for mass spectrometry have been derived from Dendral since its beginnings. Other early examples from the late 1980s include the application of neural networks to predict secondary structures of proteins^68^, the analysis of low-resolution mass spectra^69^, drug discovery^70^, or process fault diagnosis^71^ and comprehensive reviews of early ML applications before and around 1990 are provided by Burns et al.^72^ as well as Gasteiger and coworkers^73^. While data-driven regression approaches are being used e.g. for drug discovery for a long time, recent breakthroughs in ML led to significant advancements in materials/drug design^74^. These breakthroughs enabled further research directly related to light-harvesting applications, notably for the discovery of small molecules for organic light-emitting diodes^75^, and photofunctional molecules with desired excitation energies^76^ as we will outline in more detail further on in the manuscript. The versatility of data-driven techniques in the sciences can be attributed to the rich pool of different models and formulations of ML strategies, which we will briefly review in the following.

### Supervised learning

**Fig. 2:**
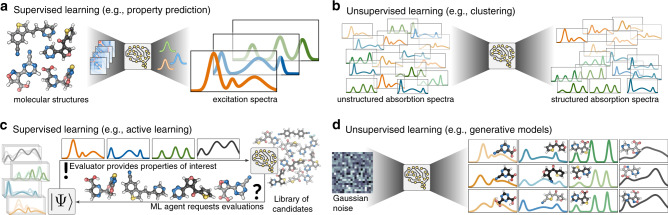
Four different variants of machine learning (ML) algorithms. **a** In supervised learning, ML models can be used to directly predict properties of interest such as absorption spectra from molecular structures. **b** Unsupervised learning methods, such as clustering can be used to identify the most relevant information in a presented dataset. **c** Active learning approaches enable a ML model to query information during the training process. **d** Generative models can simultaneously predict molecular structures and properties of interest that go beyond prespecified training sets.

One common application of ML consists in supervised learning tasks (see Fig. 2a, c). For such problems, ML models are trained to predict a set of outputs (targets) from a set of inputs (features). Hence, the models need to learn a mapping which projects given features to their associated targets, following the hidden causality of the feature-target relation. In the context of chemistry and materials science, supervised problems are encountered for example as property prediction tasks such as estimating CT excitation energies from molecular geometries. Given the molecular structures, and possibly the environment in which the structures are embedded, the ML model learns a function *f* to predict the set of desired properties, e.g. CT excitations.

During training, the model is presented with examples of (structure and environment vs. properties) pairs to infer the underlying structure-property relation. To this end, the model leverages statistical correlations from the dataset, instead of physical laws. It is important to mention that ML models for supervised tasks cannot identify or formulate dependencies of the properties on structural or environmental variables that are not included in the dataset. For example, a temperature dependence will not be discovered if temperature is not provided as one of the factors in the dataset. In fact, if the predicted properties are modulated by temperature changes, the properties of interest would be subject to a seemingly stochastic noise-level below which the prediction errors of the ML models could not be converged.

Active learning presents a special case, where labeled data is generated on-the-fly, interleaved with a prediction process and the labeled data is actively queried by the ML agent. As such, active learning relies on a closed-loop feedback mechanism and can be approached from the perspective of the supervised learning paradigm where the model actively queries a structure to be evaluated, or from the perspective of a reinforcement learning task where the model receives a reward to a set of chosen actions. Active learning is illustrated in Fig. 2c. Compared to standard supervised learning strategies, ML agents in an active learning framework do not require initial data to be trained on, which comes at the expensive of an iterative and thus less parallelizable workflow. Note, that active learning can be though of as the generalization of optimization tasks, where the difference lies in the choice of the reward function which determines the merit of each queried structure.

### Unsupervised learning

Unsupervised learning strategies, in contrast, do not aim to directly predict properties from features. Instead, their focus is on inferring the a priori probability distribution *p*, e.g. *p*(property) (see Fig. 2b). Unsupervised learning thus has the potential to reveal patterns in the provided dataset which can then be interpreted by the researcher. Examples of unsupervised strategies include clustering and anomaly detection. However, if both structures and properties are available to an unsupervised model, the joint probability distribution, *p*(property, structure), can be learned. Such unsupervised models can be understood as generative models, which predict structures and properties of molecules or materials simultaneously (see Fig. 2d). To this end, generative models typically rely on a latent space, from which properties and structures are predicted at the same time. Since both the properties and the structures are generated from the same point in the latent space, one can navigate that latent space in search for the desired property values and obtain structures directly with the expected properties. The search for a structure with desired property is formulated as a search for the point in the latent space which generates these desired properties. Since this latent space point can be decoded into a structure, both structures and properties are obtained at the same time. The inverse-design problem of finding a structure that satisfies desired properties thus no longer requires the assembly and subsequent exhaustive undirected screening of a large library of candidates. Instead, promising candidates can be identified in a more guided, and thus faster search following the structure of the latent representation.

### Representations and models

Scientific questions pose unique challenges which are not frequently encountered in the traditional sample applications of ML research. For example, molecules and materials can obey particular symmetries, such as an ambiguity in the ordering of their atoms or the invariance of properties with respect to translations and rotations. Yet, the environment of a molecule might be of particular importance and could be intrinsically disordered and challenging to describe. Different representations of the structure can highlight different aspects of a molecule or material, and thus affect the predictive power of a ML model. In fact, the identification of most informative representations, a process commonly referred to as feature engineering, has shown to be of crucial importance^77^.

Several representations have been proposed to boost the performances of ML models. One fundamental requirement on performant representations is uniqueness, i.e. the representation must be unique with respect to all relevant degrees of freedom^78^. Among the earliest developed representations are SMILES strings^79^, which encode molecules as text. Morgan fingerprints represent molecules as a bit-vector indicating the presence of molecular fragments in the molecule^80^, and typically perform well in cases where specific functional groups govern the properties of interest. Coulomb matrices and other non-topological features^81^ have been introduced in the context of learning electronic properties of molecules. They are frequently used to predict, for example, ground and excited state energies, absorption spectra or thermochemical properties. Other representations include bag of bonds/angles/dihedrals^82^, many-body tensors^83^, atom-centered symmetry functions^84^ the smooth overlap of atomic positions (SOAP)^85^, or representations based on multidimensional Gaussians (FCHL)^86^. Recently, with the rise of generative models in chemistry, other text-based representations of molecules have been proposed. Among others, GrammarVAEs^87^ and SELFIES^88^ have been suggested to increase the robustness and diversity of text-based representations. In addition, application specific representations based on hand-selected sets of microscopic properties are frequently employed^89–95^.

Novel ML models have also been specifically designed for applications in the physical sciences to intrinsically account for some of the aforementioned symmetries and unique properties of molecules and materials. Deep tensor networks (DTNNs)^96^, for example, process molecular structures based on vectors of nuclear charges and matrices of atomic distances expanded in a Gaussian basis. This encoding preserves all information relevant to the prediction of electronic properties but achieves model invariance with respect to translations and rotations. ANAKIN-ME describes an approach to develop transferable neural network potentials based on atomic environment vectors^97^. Shortly after, message passing neural networks have been introduced^98^, which interpret molecules as unstructured graphs. The recently reported TensorMol architecture uses two neural networks, one trained to account for nuclear charges and the other trained to estimate short-ranged embedded atomic energies, for the prediction of properties such as atomization energies^99^, thus extending prior work pioneered by Behler (see ref. ^100^ for an in-depth review) An extension to DTNNs has been suggested using filter-generating networks to enable the incorporation of periodic boundary conditions^101^. Only recently, inspired by the many-body expansion, hierarchically interacting particle neural networks (HIP-NN) have been developed to model the total energy of a molecule as a sum over local contributions, which are further decomposed into terms of different orders^102^. The developed representations and models provide the toolset to support studies on natural and artificial light-harvesting systems with ML, as will be demonstrated in the remainder of this perspective.

## Machine learning accelerates established workflows

Statistical methods have long been used to calibrate quantum mechanical calculations to experimental results. DFT, for example, could correctly describe the quantum nature of matter if the exact exchange-correlation functional was known and a complete basis-set was used. In reality, DFT relies on approximative exchange-correlation functionals, which determine the accuracy of DFT-based property predictions^103^. It has, therefore, long been of interest to calibrate DFT-computed properties to experimental results with simple statistical models such as linear regression, for example in the context of predicting the 1H NMR shielding tensor^104^, or pKa values^105^. More elaborate neural network models have been used to predict corrections to molecular energies obtained with smaller basis sets based on results obtained with larger basis sets^106^.

In TD-DFT calculations, exchange functionals tend to underestimate CT excitations due to self-interaction errors^107,108^. Potential energy surfaces estimated with TD-DFT can, therefore, be incorrect, which complicates excited state dynamics calculations. Yet, TD-DFT provides a relatively inexpensive alternative to obtain excited state properties compared to computationally more involved schemes, such as GW, EOM-MP2, EOM-CC, RPA or Full CI. As such, empirical corrections to TD-DFT results which alleviate the accuracy shortcomings without substantially increasing the computational demand like the aforementioned approaches have long been of interest, particularly to compute properties of candidate materials for solar cell applications. One example consists in the in silico estimation of the reachable open-circuit voltage, which corresponds to the maximum voltage available from a solar cell and is fundamentally limited by the bandgap of the candidate material. Notably, the Harvard Clean Energy Project (CEP) has shown that linear regression provides a robust method to calibrate the open-circuit voltage of photovoltaic devices to accurately reproduce experimental results at the  <30 meV level^109^. The database generated from the CEP results^110^ inspired further studies on the calibration of excitation energies with more complex models such as neural networks^111^, as well as the development of molecular descriptors for the prediction of power conversion efficiencies^112^. In addition, neural networks and support vector machines have been used for more than a decade to improve the accuracy of TD-DFT predictions, e.g. to predict absorption energies of small organic molecules^113,114^.

With the increasing availability of datasets, ML experienced a steep rise in interest for electronic structure predictions in the last years. Initial studies focused on the direct prediction of ground state properties, such as atomization energies from Coulomb matrices using kernel ridge regression^81^. Promising prediction accuracies encouraged attempts to predict excited state properties shortly after^115^. To systematically study and compare the performance of neural network models, a benchmark set consisting of all stable, synthetically accessible organic molecules with at most seven heavy atoms, referred to as QM7, was introduced^115^. More extensive benchmark sets followed, such as the QM9 dataset^116^ as well as MoleculeNet as a collection of several datasets^117^. These benchmark sets enabled more thorough investigations of the applicability of ML models to calibrate inexpensive approximate quantum methods to more accurate calculations and experiments: a variety of thermochemical properties including enthalpies, free energies, entropies and electron correlations have been predicted for small molecules^118^; hierarchical schemes based on multilevel combination techniques have been introduced to combine various levels of approximations in quantum chemistry with machine learning; chemical shifts in NMR have been prediced with kernel ridge regression^17^ and the SOAP kernel^119^; and ground state properties could be predicted at systematically lower errors than DFT calculations^120^. The observed prediction accuracies, therefore, suggest that ML could indeed complement DFT as one of the most popular electronic structure approaches.

One step further, ML can also aid in the prediction of excitation spectra. It has been demonstrated that kernel ridge regression can be applied to calibrate electronic spectra obtained from TD-DFT calculations to CC2 accuracies in a Δ-learning approach^121^. The possibility to directly predict excitation spectra from molecular structures has also been reported^122^. Specifically, simple feed-forward neural network models were used to predict the positions and the spectral weights of peaks in molecular ionization spectra for small organic molecules encoded as Coulomb matrices. The prediction accuracies could be further improved with more elaborate convolutional neural network models and DTNNs. With the accurate prediction of excitation spectra, optical properties such as band gaps can be readily estimated for novel solar cell candidates, which accelerates the search for promising materials.

### Prediction of dynamics

Studying the behavior of light-harvesting molecules and materials under operating conditions, including interactions with light, radiative, and non-radiative decay processes as well as degradation processes, requires highly accurate modeling of these systems over long time scales. However, computational modeling of large systems bridging orders of magnitude in time poses extreme challenges to conventional simulation methods. Molecular dynamics simulations enhanced with ML based force fields promise to accelerate simulations of molecular materials as well as crystalline materials and can provide deeper mechanistic insights. First molecular dynamics simulations with a purely ML-based ground state density functional have recently been reported^123^. This study demonstrates how electron-densities can be predicted from approaches similar to kernel ridge regression and was used to time-evolve small molecular systems in their ground states. Ground-state molecular dynamics simulations have also been realized with Gaussian process regression, where forces are either predicted directly by the regressor or computed on-the-fly from DFT calculations^124^. This active learning strategy to build an accurate ML model on-the-fly for MD simulations has further been demonstrated in the context of amorphous and liquid hafnium dioxide^125,126^, and aqueous sodium hydroxide^127^.

Moreover, ML has also been used for excited state predictions to study the dynamics of excitons in natural light-harvesting complexes. For example, the acceleration of exciton dynamics calculations with feedforward neural networks has been reported for the FMO pigment–protein complex^128^. Furthermore, EET characteristics of nature-inspired excitonic systems have been estimated with neural network models, thus reducing the computational cost of computationally involved methods for open quantum systems dynamics^129^. Recently, the on-the-fly construction of potential energy surfaces for non-adiabatic excited state dynamics calculations with kernel ridge regression has been reported for selected molecules^91^. While demonstrating that excited state dynamics calculations can indeed be accelerated with ML techniques, the predicted potential energy surfaces initially showed large deviations in the vicinity of conical intersections, which required corrections from additional QM calculations. Deep-learning models have been suggested to alleviate this bottleneck and perform pure ML-based excited state dynamics calculations^95,130^. As the aforementioned studies only focused on selected molecules, transferability of the models to more diverse molecule classes has yet to be demonstrated. Nonetheless, these studies demonstrate that ML emerges as a promising tool to enable large-scale excited-state dynamics studies. Such tools can allow for detailed mechanistic studies of EET and CT processes in light-harvesting devices at the atomic level. For example, the behavior of complex perovskite structures could be investigated under operating conditions to observe effects such as the formation of ferroelectric domains or the migration of defects and ions on a microscopic scale. These observations could, in turn, inspire the design of more robust and long-lived devices.

## Machine learning can transform established workflows

While the design and fabrication of light-harvesting devices require a profound understanding of EET and CT processes, further aspects need to be considered to design economically viable solutions. For example, the solubility and photostability of materials candidates and the cost to synthesize them are keys for a more complete and comprehensive description. However, computing such properties with ab initio approaches poses major challenges, as precise estimates can at best be obtained at high computational costs. Since ML leverages statistical correlations, rigorous physical descriptions of considered phenomena are not needed to quantify such secondary materials aspects. For example, a recent study demonstrated the construction of a data-driven model to estimate Hansen solubility parameters in two different approaches^90^: one constructed model was based on molecular properties including sigma profiles, electrostatics, geometric and topological parameters evaluated with semi-empirical and DFT methods, while the other model only relied on inexpensive topological parameters (molecular fingerprints). Both models, based on Gaussian processes which are well suited for small and noisy datasets, were found to be similarly accurate in their prediction, despite the more diverse data available to the first model. Instead, relevant statistical correlations could be identified from the topological features alone, resolving the need for electronic structure calculations and thus accelerating the solubility parameter estimation by about 720x.

The potential of ML to assess device performances directly from a set of features describing materials properties has been demonstrated for the prediction of experimental power conversion efficiencies (PCEs) of small molecule OPVs^92^. Accurate theoretical PCE predictions require high-level quantum chemistry calculations to correctly account for all influential effects such as electron–electron interactions and electron–phonon couplings. Instead of accelerating quantum chemical approaches, gradient boosting models were used to directly predict PCEs from a set of 13 hand-picked microscopic molecular properties, which were known or hypothesized to affect the energy conversion process. Another study has shown that design principles for acceptor molecules in OPVs can be derived from a Gaussian process based calibration model^131^. Bandgaps of hybrid organic-inorganic perovskites (HOIP) have also been predicted directly with several ML approaches^132^. Initially, 30 properties were selected as features for each of the HOIP candidates, and the benefit of each descriptor to improve the predictive power of a gradient boosting regression was assessed. A subset of 14 features was then identified, where the tolerance factor, calculated from the ratio of ionic radii, was revealed to be the most influential descriptor. Both studies demonstrate the applicability of ML methods to estimate materials performances from a set of low-level descriptors without the need of extensive quantum chemistry calculations. Moreover, specific microscopic materials properties have been identified to be particularly influential, which can be used to derive design principles.

### Unsupervised strategies for light-harvesting

Similarly to property predictions with supervised learning, the application of unsupervised strategies to light-harvesting is an active field of research. In fact, unsupervised strategies have recently been proposed to aid in the optimization of multi-junction solar cells toward maximized yearly energy yields instead of maximized efficiencies at standard conditions^133^. Specifically, the cost-effective computation of the yearly energy yield from a set of reference solar spectra has been enabled by identifying the most informative subset of characteristic spectra via k-means clustering. Further, clustering strategies have been used to derive design principles for organic semiconductor design^134^, which enabled the in silico discovery of molecular crystals with improved charge-transfer properties.

In addition to clustering strategies, generative models are applied for the de novo design of novel materials for light-harvesting. Variational autoencoders (VAEs) have first been suggested for automatic chemical design^135^, and demonstrated on the generation of drug-like molecules and molecules for organic light-emitting diodes. Extensions to this work improved the validity of the generated molecules by adapting the SMILES-based molecule encoding, leading to e.g. grammar VAEs^87^. These extended encodings have recently been employed to generate organic donor–acceptor candidates for polymer solar cells^94^. The authors demonstrate that grammar VAEs can be efficiently applied when modifying the standard SMILES representation for a donor–acceptor molecule into a more coarse grained representation, where individual structural features are highlighted. While the introduced coarse grained representation narrows the search space, it also enables the grammar VAE to focus on relevant and promising molecules. Starting from a randomly selected training set of candidate polymers where only 11% of the polymers satisfy the chosen thresholds for LUMO levels and optical gaps, the authors demonstrate that a grammar VAE can be trained to generate promising polymer candidates at a rate of 61%.

The ChemTS library implements de novo molecular design by combining Monte Carlo tree searches with recurrent neural networks (RNN)^136^. ChemTS generates a shallow tree of incomplete text-based molecule encodings, and completes each branch with a RNN. The most promising branches are kept, while the others are discarded. Following this strategy, the discovery of five photofunctional molecules with desired lowest excitation levels has recently been reported^76^. This study iteratively generated organic molecules and computed their excitation energies with TD-DFT in a closed-loop approach. Notably, out of the six molecule candidates discovered from this in silico screening, the expected properties could be experimentally verified for five of them.

### Synthesis and fabrication planning

Although generative models have the ability to propose novel molecules and material candidates in silico, synthesis pathways or fabrication protocols might not be known. Thus, one of the main challenges for generative models is to ensure the transferability of computationally predicted lead candidates to synthesis and experiment. To alleviate this obstacle, quantitative estimates of the synthesizability can be included as targeted properties during the design process. Synthesizability is typically quantified via heuristics based on domain expertise or ground truth data, and multiple approaches have been developed^137–139^. Recently, an improved synthesizability score based on a neural network model trained on 12 million reactions has been suggested^140^, targeting the inexpensive indication of synthetic accessibility of a compound. The development of ML tools for the direct prediction of chemical syntheses, either via forward synthesis or retrosynthesis techniques, has also experienced increased attention^141,142^. Yet, fast experimental verifications of the computationally predicted lead candidates might still not be possible due to additional hardware and reagent constraints^3^. Instead, aspects about the synthesizability and the feasibility of selected synthesis pathways could be taken into consideration when generating a large library of realizable candidate materials. For example, coarse-grained molecular representations can encode molecules for which the synthesis path is known ahead of time. Active learning approaches can then be applied to navigate the search space for the most promising candidate, knowing how to synthesize every element of the search space.

## Discussion and outlook

**Fig. 3:**
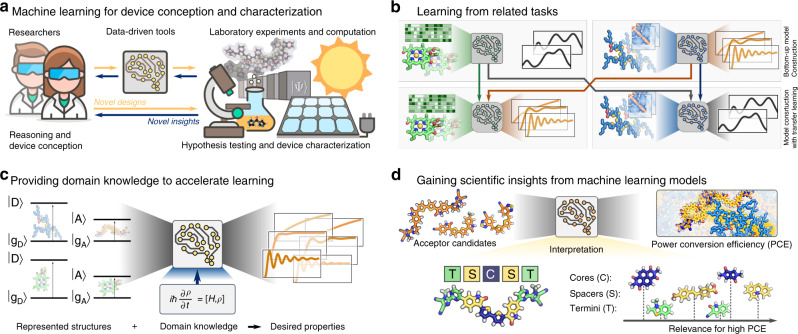
Future directions to explore machine learning (ML) applications for light-harvesting. **a** ML emerges as a technology that can be used for both the conception and the characterization of light-harvesting devices by amplifying state-of-the-art technologies to enable more versatily discovery workflows with higher throughput at lower cost. **b** Transfer learning can enable accelerated ML predictions at lower data requirements by leveraging information learned from related tasks. **c** Providing domain knowledge, such as fundamental laws of physics that cannot be violated in the considered light-harvesting system can aid in the training of ML models. **d** Interpreting trained ML models can help to conceptualize empirical findings and formulate scientific insights, illustrated on the example of constructing non-fullerene acceptor candidates from core (C), spacer (S), and terminal (T) fragments to improve the power conversion efficiency (PCE).

Data-driven approaches are emerging as versatile and viable technology for light-harvesting research, connecting complete and comprehensive bottom-up theoretical models with Edisonian trial-and-error strategies. Indeed, light-harvesting research, notably for the design of OSCs and PSCs, increasingly integrates data-driven tools into their workflows at various levels^143^, including property screening^144,145^, candidate selection^76,136^, analysis^146^, and interpretation^89,147^. We have highlighted some of the ML models specifically designed to find new light-harvesting materials^76,94,135,136^. The development of light-harvesting technologies is an elaborate process, which involves design choices based on theoretical models and hypotheses regarding the governing principles of light-harvesting, and the synthesis and characterization of light-harvesting materials and devices. With the capacity to infer causal structure-property relations from experiments and collected data, the applicability of ML can be found on both ends, device conception, and device testing, where the boundaries of state-of-the-art technologies can be pushed even further with data-driven approaches (see Fig. 3a).

While ML has been shown to expedite both the theoretical and phenomenological understanding of the fundamental processes around light-harvesting, the applicability of an individual ML model is still mostly limited to specific aspects, and models need to be trained from the ground up for every new application. Image recognition has emerged as an example where parts of trained ML models can be reused when moving to a different application, such that patterns learned in previous studies are carried over to accelerate the learning process for new applications. Since light-harvesting applications are governed by the same fundamental quantum mechanical phenomena, transfer learning approaches could provide a more comprehensive picture of the structure-property relations of light-harvesting systems (see Fig. 3b).

The remarkable successes of ML in modeling highly complex physical relations in light-harvesting applications have mostly been achieved with large datasets, assembled and collected with a lot of effort^76,92,94^. Structure-property relations have been constructed based on statistical correlations contained in these datasets. Apart from possible limitations arising from limited representations of the physical reality, and the resulting selection bias inherent to the datasets, the many computations or experiments required to assemble the datasets often cause substantial costs. A more sustainable approach, alleviating the data requirements, consists in active learning strategies, where ML models are enabled to actively incorporate new data in a closed-loop process. Yet, even with active learning, the models’ predictions solely rely on statistical correlations. Providing domain knowledge, i.e. leveraging the scientific understanding of light-harvesting to date, has the potential to substantially lower the data requirements (see Fig. 3c). For example, it has been demonstrated that neural networks can be trained to predict the heights of objects under gravity from images and Newton’s equations of motion, but without being provided explicitly labeled data^148^. Thus, ML models could be supplied with the relevant known laws of physics, which are impossible to be violated in the considered light-harvesting systems, and complete their interpretations of structure-property relations based on provided (or queried) examples. Such approaches have recently been demonstrated in the context of quantum chemistry calculations, where data-driven approaches by construction encode the physics of valid wave functions^149,150^.

Similarly, the statistical correlations identified by ML models provide opportunities for interpretations and the formulation of novel scientific concepts (see Fig. 3d). The derivation of design principles by analyzing the architecture of trained ML models has already been demonstrated for feature engineering and applications in light-harvesting, specifically on the prediction of PCEs from molecular descriptors^92^. However, trained ML models can also be used as an investigation tool for the inexpensive testing of hypotheses, which has recently been demonstrated in the context of predicting the timescales of the chemiluminescent decomposition of small molecules^89^. Yet, these studies where ML technologies are used to gain scientific understanding present only isolated cases, as most existing ML models are not intrinsically interpretable but have focused on high prediction accuracies. Data-driven approaches need to generally transition from predictive models to explaining models to contribute to inspire insights and drive light-harvesting research further. We, therefore, consider the development of interpretable ML models for a large range of applications in light-harvesting research to be one of the outstanding challenges to advance the field.

Succeeding in these endeavors enables opportunities to gain insights into the challenging scientific questions around light-harvesting, such as understanding the interplay between microscopic features and mesoscale properties of natural pigment–protein complexes, the quantum mechanical effects modulating non-radiative decay rates in OSCs, or the influence of processing conditions on the stabilities and efficiencies of perovskite materials. These phenomena are only a few examples of the complex processes involved in light-harvesting, and are challenging to approach with conventional computational and experimental tools. The data-driven nature of ML models provides the opportunity to amplify the cutting edge theoretical models and experimental technologies for more elaborate and more efficient discovery workflows, which are emerging recently emerging in the field. The transition to predictive, intuitive, and interpretable ML models to complement theoretical studies and experimentation have the potential to provide important scientific insights and inspire design rules to improve materials, processing conditions, and eventually device properties of light-harvesting technologies.
